# Analysis of nine compounds from *Alpinia oxyphylla* fruit at different harvest time using UFLC-MS/MS and an extraction method optimized by orthogonal design

**DOI:** 10.1186/1752-153X-7-134

**Published:** 2013-08-02

**Authors:** Yong-Hui Li, Feng Chen, Jun-fang Wang, Yong Wang, Jun-Qing Zhang, Tao Guo

**Affiliations:** 1 Hainan Provincial Key Laboratory of R&D on Tropical Herbs, Hainan Medical University, Haikou 571199, China; 2 School of Pharmacy, Hainan Medical University, Haikou, Hainan 571199, China; 3 School of Life and Engineering, Lanzhou University of Technology, Lanzhou 730050, China

**Keywords:** UFLC-MS/MS, *Alpinia oxyphylla* fruits, Quality control

## Abstract

**Background:**

The dried fruits of *Alpinia oxyphylla* Miq have been widely used as an herbal medicine for the treatment of diarrhea and enuresis in China. Medicinal chemistry studies revealed that the tepenes, diphenylheptanes and flavones were the main components. Therefore, these three kinds of components should be chosen as the bioactive marker compounds for the quality control of *A. oxyphylla* fruits. Moreover, multiple active components has been widely recognized to be a more feasible method for the quality control of herbal medicines. This study firstly provided a better method for comprehensive component analysis of *A. oxyphylla* fruits. Meanwhile, the different harvest time was also evaluated.

**Results:**

The solvent-to-sample ratio was the most important factor comparing with solvent, extraction time and temperature. The highest yield of nine compounds was achieved with 70% ethanol-water and a solvent-to-sample ratio of 20:1 at 60°C for 30 min. The optimized analytical method for ultra fast high performance liquid chromatography (UFLC) was a gradient elution using water containing 0.04‰ formic acid (A) and methanol containing 0.04‰ formic acid (B), at a flow rate of 0.3 mL/min. Using this optimized method, nine compounds were simultaneously separated and quantified by UFLC coupled with tandem electro-spray ionized mass spectrometry (MS/MS).

**Conclusions:**

The contents of the six bioactive compounds were reported in *A. oxyphylla* for the first time. The contents of nine compounds of different harvest time fruits of *A. oxyphylla* were assessed under the optimized extraction and UFLC-MS/MS analytical conditions, and the 45-day culture fruit had the highest content levels.

## Background

*Alpinia oxyphylla* fruit, Yizhi in Chinese, is the dried fruit of *A. oxyphylla* Miq., which has been widely used as an herbal medicine in China for the treatment of diarrhea and enuresis [1]. Pharmacological investigations have shown that the fruits of *A. oxyphylla* possess an array of activities, including anti-inflammatory [2,3], anti-allergy [4], anti-ulcer [5] and neuroprotective [6-8] activities. It was reported that the tepenes, diphenylheptanes and flavones were the main components in *A. oxyphylla* extract [9,10]. Therefore these three kinds of components should be chosen as the bioactive marker compounds for the quality control of *A. oxyphylla* fruits.

So far, some studies on the quality evaluation of *A. oxyphylla* fruits by high performance liquid chromatography (HPLC) [11,12] have been reported. However, the reported HPLC profiles could not achieve satisfactory resolution and the researches only focused on the nootkatone of *A. oxyphylla* fruits. As a result, other compounds’ quantification has not been reported. Now, quantitative analysis of multiple active components has been widely recognized to be a more feasible method for the quality control of herbal medicines [13,14]. Therefore, we developed and validated a simple UFLC-MS/MS method for the simultaneous analysis of the nine compounds of *A. oxyphylla* fruits from various harvest time. This study firstly provided a better method for comprehensive component analysis of *A. oxyphylla* fruits. Meanwhile, the different harvest time of the herb for quality evaluation were also discussed.

## Experimental

### Chemicals and materials

**Figure 1:**
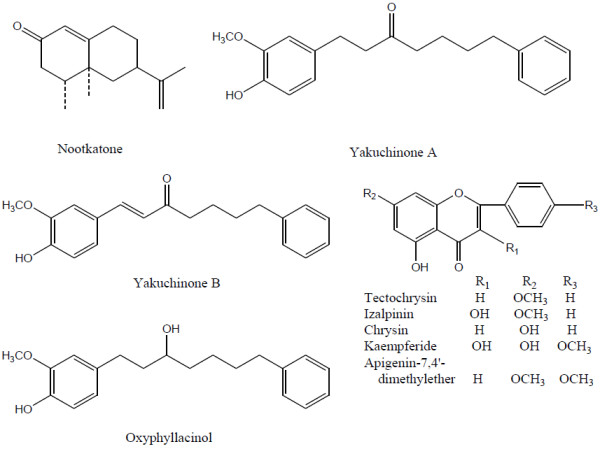
Chemical structures of the nine phytochemicals.

The dried fruits of *A. oxyphylla* of different harvest time (10-day, 15-day, 20-day, 25-day, 27-day, 30-day, 35-day, 40-day, 45-day, 50-day, 55-day, 60-day and 65-day) were collected from Qiongzhong county, Hainan province, China. The material was identified by vice Prof. Jian-ping Tian of Hainan Medical University. Reference standards of nootkatone, yakuchinone A, yakuchinone B, oxyphyllacinol, tectochrysin, izalpinin, chrysin, kaempferide and apigenin-7,4′-dimethylether were isolated and purified in our laboratory. On the basis of UV, NMR and MS analysis, the structures of isolated reference standards were confirmed, and their purities determined using HPLC–PDA-MS were over 98.0%. Their structures are presented in Figure 1. Methanol was HPLC-grade from Merck (Darmstadt, Germany) and deionized water was purified by a Cascada IV super purification system (Pall Corporation, NY, USA). Other reagent solutions were analytical grade (Shanghai Chemical Reagent Company, Shanghai, PR China).

### Preparation of standard solutions

A mixed standard stock solution containing nootkatone (**1**), yakuchinone A (**2**), yakuchinone B (**3**), oxyphyllacinol (**4**), tectochrysin (**5**), izalpinin (**6**), chrysin (**7**), kaempferide (**8**), and apigenin-7,4'-dimethylether (**9**) was prepared in acetonitrile. The working standard solutions were prepared by diluting the mixed standard solution with acetonitrile to a series of proper concentrations within the ranges: **1**, 2.00-1000.00 ng/mL; **2**, 1.00-1000.00 ng/mL; **3**, 1.00-1000.00 ng/mL; **4**, 5.83-1165.00 ng/mL; **5**, 2.07-1135.00 ng/mL; **6**, 5.00-1000.00 ng/mL; **7**, 2.00-1000.00 ng/mL; **8**, 2.16-1080.00 ng/mL; **9**, 5.00-1000.00 ng/mL. The standard stock and working solutions were all stored at -40°C until use and filtered through a 0.22 μm membrane prior to injection.

### Preparation of sample solutions

Thirteen batches fruits of *A. oxyphylla* were ground into powder and sieved with a 40 mesh, respectively. An aliquot (0.5 g) was weighed precisely and refluxed with 10 ml of 70% ethanol for 30 min at 60°C, and finally made to a volume of 10 ml using 70% ethanol. Three replicates of the extraction process were carried out on the independent samples. The solution was filtered through 0.22 μm membrane prior to use. The filtrates were diluted one hundred times with methanol, and a 5 μl aliquot was injected into the UFLC-MS/MS system for analysis.

### Apparatus and operation conditions

#### Liquid chromatography

Chromatographic analysis was performed on a Shimadzu LC-20 AD UFLC system (Shimadzu Corp., Tokyo, Japan), consisting of a binary pump solvent management system, an online degasser, and an autosampler. A Shim-Pack XR-ODS column (100 mm × 2.0 mm, 2.2 μm) was employed and the column temperature was maintained at 30°C. The mobile phase was composed of A (water containing 0.04‰ formic acid) and B (methanol containing 0.04‰ formic acid) using a gradient elution of 2% B at 0 min to 100% B at 10 min with a flow rate set at 0.30 mL/min. The injection volume was 5 μL.

#### Mass spectrometry

An AB-SCIEX API 4000^+^ mass spectrometer (Toronto, Canada) interfaced via a Turbo V ion source with a Shimadzu Prominence UFLC chromatographic system (Shimadzu Corporation, Kyoto, Japan). The AB-SCIEX Analyst software packages were used to control the UFLC-MS/MS system, as well as for data acquisition and processing. The mass spectrometer was operated in the positive ion ESI mode with multiple reaction monitoring (MRM) for all the analytes. The pneumatically nebulized ESI spraying was achieved by using inner coaxial nebulizer N_2_ gas of 55 psi through a TurboIonSpray probe, a high voltage of + 5.5 kV applied to the sprayer tip, and heated dry N_2_ gas of 55 psi at 550°C from two turbo heaters adjacent to the probe. To prevent solvent droplets from entering and contaminating the ion optics, a curtain N_2_ gas of 25 psi was applied between the curtain plate and the orifice. The collision gas (N_2_) flow was set at level 4. The parameters for MRM were optimized for each analyte and selected values are given in Table 1.

**Table 1:** **Retention time (t**_R_**) and parameters for MRM of compounds used in this study**

**Compounds**	**t**_**R **_**(min)**	**[M + H]**^**+ **^**(m/z)**	**Quantitative ion (m/z)**	**Scan time (msec)**	**DP (volts)**	**EP (volts)**	**CE (volts)**	**CXP (volts)**
Nootkatone	10.98	219.2	163.0	25	80	10	22	11
Yakuchinone A	10.59	313.2	136.9	25	80	10	13	8
Yakuchinone B	10.75	311.2	117.0	25	80	10	30	11
Oxyphyllacinol	10.58	315.3	137.0	25	22	10	22	11
Tectochrysin	10.97	269.1	226.0	25	106	10	43.5	11
Izalpinin	11.12	285.0	242.0	25	110	10	43	11
Chrysin	10.05	255.1	152.9	25	110	10	42	10
Kaempferide	10.22	301.1	286.0	25	110	10	37	18
Apigenin-7,4^′^-dimethylether	11.03	299.2	256.0	25	110	10	45	14

### Method validation

#### Calibration curves, limits of detection (LOD) and quantification (LOQ)

A series of concentrations of standard solution were prepared for the establishment of calibration curves. The peak areas were plotted against the corresponding concentrations to obtain the calibration curves. LODs and LOQs were determined using diluted standard solution when the signal-to-noise ratios (S/N) of analytes were about 3 and 10, respectively. The S/N was calculated as the peak height divided by the background noise value.

#### Precision, repeatability and accuracy

The intra-day and inter-day variations, which were chosen to determine the precision of the developed method, were investigated by determining the 9 analytes in six replicates during a single day and by duplicating the experiments on three consecutive days. Variations of the peak area were taken as the measures of precision and expressed as percentage relative standard deviations (RSD).

Repeatability was confirmed with six independent analytical sample solutions prepared from the same batch of sample (the fruits of *A. oxyphylla* collected at 55-day) and variations were expressed by RSD. One of the sample solutions mentioned above was stored at 25°C, and injected into the apparatus at 0, 2, 4, 8, 12 and 24 h, respectively, to evaluate the stability of the solution.

A recovery test was used to evaluate the accuracy of this method. The test was performed by adding known amounts of the 9 standards at low (80% of the known amounts), medium (the same as the known amounts) and high (120% of the known amounts) levels. The spiked samples were then extracted, processed and quantified in accordance with the aforementioned methods. The average recovery percentage was calculated by the formula: recovery (%) = (observed amounts ‒ original amounts)/spiked amounts × 100%.

### Identification and quantification

Identification of the target peaks was performed by comparing their UFLC retention times, and mass/charge ratios (m/z) with those of the standards. In order to further confirm the structures of the constituents, standards and samples were analyzed by UFLC–MS/MS in positive ion modes. Quantification was performed using linear calibration plot of peak area and concentration.

### Optimization of compounds extraction

The 55-day fruit was used to optimize the extraction condition of nine compounds. The solvent, solvent-to-sample ratio, extraction time and extraction temperature were optimized. The powder of 0.5 g of the fruit was extracted with two solvents, methanol or ethanol, at 100% and 70% aqueous solution (v/v). Four solvent to sample ratios [10:1, 20:1, 40:1, and 80:1, (v/w)] and four extraction time (30 min, 60 min, 90 min, and 120 min) were tested at two temperatures (60°C or 90°C). All the factors were investigated using an orthogonal (L_16_4^3^ × 2^1^) experimental design, and each extraction was tested in triplicate.

Using the selected optimal extraction conditions, the different harvest time fruits of 0.5 g were accurately weighed and extracted at 60°C with 10 mL 70% ethanol for 30 min. The extraction of each sample was performed in triplicate. And the solution was filtered through a 0.22 μm filter before UFLC-MS/MS analysis.

## Results and discussion

### Optimization of the extraction conditions

The parameters obtained from the orthogonal (L_16_4^3^ × 2^1^) test of three types of compounds extraction were weighed and quantitatively analyzed using evaluation indices *k* and *R* (Table 2). The results showed that the *R* value of factor B was the highest for the nootkatone and diphenylheptanes ingredients, and less significant for the flavones components. This indicated the solvent-to-sample ratio was the most important factor among the four parameters. Extraction time and extraction temperature generally had much less significant effects on the three types of ingredients. Similarly, solvent was important for all the three types of ingredients. Based on the *R* values, the factors could be ranked by importance for the overall three types of ingredients as follows: solvent-to-sample ratio > solvent > extraction time > extraction temperature.

**Table 2:** **Orthogonal (L**_**16**_**4**^**3**^ **× 2**^**1**^**) extraction efficiency results**

**Test no.**	**A (solvents)**	**B (Solvent to sample ratio)**			**C (Time)**		**D (Tempetature)**		**Yields (mg/g)**			
									**Nootkatone**	**Diarylheptanes**		**Flavones**
1	A1 (100% MeOH)	B1 (20:1)			C1 (30 min)		D1 (60°C)		1.420	1.429		0.095
2	A1 (100% MeOH)	B2 (40:1)			C2 (60 min)		D1 (60°C)		1.391	1.378		0.087
3	A1 (100% MeOH)	B3 (60:1)			C3 (90 min)		D2 (90°C)		1.213	1.266		0.087
4	A1 (100% MeOH)	B4 (80:1)			C4 (120 min)		D2 (90°C)		1.196	1.320		0.075
5	A2 (70% MeOH)	B1 (20:1)			C2 (60 min)		D2 (90°C)		1.258	1.287		0.067
6	A2 (70% MeOH)	B2 (40:1)			C1 (30 min)		D2 (90°C)		1.272	1.371		0.079
7	A2 (70% MeOH)	B3 (60:1)			C4 (120 min)		D1 (60°C)		1.172	1.295		0.074
8	A2 (70% MeOH)	B4 (80:1)			C3 (90 min)		D1 (60°C)		1.262	1.375		0.079
9	A3 (100% EtOH)	B1 (20:1)			C3 (90 min)		D1 (60°C)		1.256	1.401		0.076
10	A3 (100% EtOH)	B2 (40:1)			C4 (120 min)		D1 (60°C)		1.253	1.375		0.068
11	A3 (100% EtOH)	B3 (60:1)			C1 (30 min)		D2 (90°C)		1.157	1.313		0.079
12	A3 (100% EtOH)	B4 (80:1)			C2 (60 min)		D2 (90°C)		1.128	1.243		0.069
13	A4 (70% EtOH)	B1 (20:1)			C4 (120 min)		D2 (90°C)		1.427	1.543		0.092
14	A4 (70% EtOH)	B2 (40:1)			C3 (90 min)		D2 (90°C)		1.314	1.396		0.078
15	A4 (70% EtOH)	B3 (60:1)			C2 (60 min)		D1 (60°C)		1.304	1.339		0.076
16	A4 (70% EtOH)	B4 (80:1)			C1 (30 min)		D1 (60°C)		1.216	1.402		0.082
	Yield of Nootkatone				Yield of Diarylheptanes				Yield of Flavones			
	A	B	C	D	A	B	C	D	A	B	C	D
K1	1.305	1.341	1.267	1.284	1.348	1.415	1.379	1.374	0.086	0.083	0.084	0.080
K2	1.241	1.308	1.270	1.246	1.332	1.380	1.312	1.342	0.075	0.078	0.075	0.078
K3	1.199	1.212	1.261		1.333	1.303	1.360		0.073	0.079	0.080	
K4	1.315	1.201	1.262		1.420	1.335	1.383		0.082	0.076	0.077	
Rb	0.117	0.140	0.009	0.039	0.088	0.112	0.072	0.032	0.004	0.006	0.009	0.001
Important	B > A > D > C				B > A > C > D				C > B > A > D			
Order optimal level	A4	B1	C2	D1	A4	B1	C4	D1	A1	B1	C1	D1

Solvent-to-sample ratio of 20:1 gave a higher yield for all the three types of ingredients than other ratios. The effect of extraction solvent was slightly different for three types of components. The ethanol-water (70:30) solvent gave the highest yields for nootkatone and diphenylheptanes, followed by flavones. Extraction temperature of 60°C gave the highest yields for all the three types of ingredients. Extraction time was a bit complicated factor for the yields of three types of components, but it had less effect on the yields of three ingredients. So the 20:1 of 70% ethanol (v/v) for 30 min at 60°C was chosen as the optimized condition for the extraction of nine compounds from *A. oxyphylla.*

### Optimization of the chromatographic and mass spectrometric conditions

In order to achieve optimum separation in a short analysis time, the chromatographic conditions such as column, mobile phase and gradient program were optimized in the preliminary test. Two brands of analytical columns, Shim-Pack XR-ODS column (100 mm × 2.0 mm, 2.2 μm) and Agilent Zorbax (100 mm × 4.6 mm, 1.8 μm), were compared. The results showed the former one obtained chromatograms with better resolution of adjacent peaks within shorter time. Although all compounds could be separated satisfactorily with the Agilent Zorbax, it had a longer retention time. Therefore, the Shim-Pack XR-ODS column was selected as analytical column. In this study, various mobile phase, methanol–water, acetonitrile-water, acetonitrile-alkaline aqueous solution and acetonitrile-acid aqueous solution were tested for good chromatographic behavior and appropriate ionization. Acetonitrile-formic acid solution was finally selected. The concentration of formic acid was further optimized to be 0.04‰. The column temperature was set at 30°C, rather than 25°C or 35°C.

**Figure 2:**
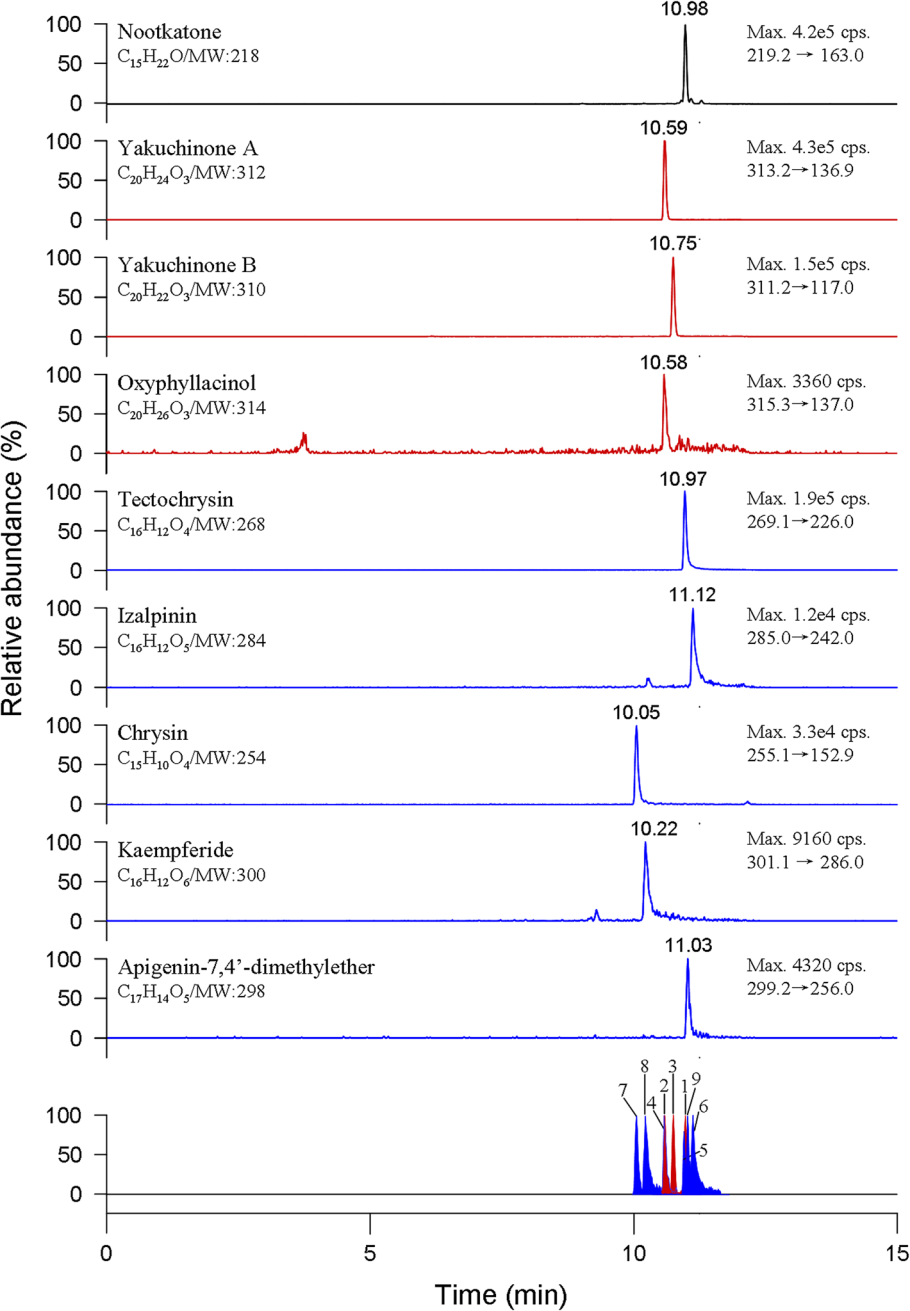
**Typical LC-MS/MS chromatograms of nine phytochemicals from *A. oxyphylla *fruit sample.** They were identified by the comparison of the retention time and mass spectra (MRM mode) with the corresponding pure compounds.

MS spectra were studied in both positive and negative ion modes. It was found that compared to the negative ion mode, yakuchinone A and yakuchinone B had higher sensitivity in the positive ion mode, which made it easier to detect their contents in *A. oxyphylla* fruits, and easier to confirm molecular ions or protonated molecule ions in the identification of each peak. As such, the positive MS ion mode was selected. The chemical structures of nine components were characterized based on their retention behaviors and MS information, such as quasi-molecular ions [M + H]^+^ and fragment ions. The ion pairs of precursor-to-product for MRM detection were generated by the Compound Optimization which was embedded in the Analyst software. Each compound was investigated individually to achieve optimal scan time, DP, EP, CE and CXP. Under the optimized UFLC and MS/MS conditions, all nine compounds in the fruits of *A. oxyphylla* were identified and quantified. Retention time and MS information for each analyte including [M + H]^+^, quantitative ions, scan time, DP, EP, CE and CXP are shown in Table 1, and UFLC–MS/MS chromatograms of 9 markers in Figure 2.

### Analytical method validation

The proposed UFLC-MS/MS method for quantitative analysis was validated by determining the linearity, LOD, LOQ, intra-day and inter-day precisions, stability and accuracy. As shown in Table 3, all calibration curves showed good linearity (r > 0.9885) within the test ranges, and the overall LODs and LOQs were in the range of 0.60-1.75 ng/mL and 2.00-5.83 ng/mL, respectively. The RSD values of intra- and inter-day variations, repeatability and stability of the nine analytes were all less than 5% (Table 4). The overall recoveries lay between 95.1% and 106% with RSD less than 5.63%. All the results mentioned above indicated that the established method was accurate.

**Table 3:** Regression data of nine compounds

**Compounds**	**Range (ng/mL)**	**Linear regression equation**	**Correlation coefficient**	**LOD (ng mL**^**−1**^**)**	**LOQ (ng mL**^**−1**^**)**
Nootkatone	2.00-1000.00	Y = 5910X + 2790	0.9979	0.60	2.00
Yakuchinone A	2.00-1000.00	Y = 7090X + 12100	0.9997	0.60	2.00
Yakuchinone B	2.00-1000.00	Y = 8100X + 6050	0.9901	0.60	2.00
Oxyphyllacinol	5.83-1165.00	Y = 763X + 1130	0.9903	1.75	5.83
Tectochrysin	2.07-1135.00	Y = 16300X + 8780	0.9973	0.62	2.07
Izalpinin	5.00-1000.00	Y = 2160X + 2190	0.9908	1.50	5.00
Chrysin	2.00-1000.00	Y = 5010X + 3010	0.9919	0.60	2.00
Kaempferide	2.16-1080.00	Y = 1040X + 1470	0.9885	0.65	2.16
Apigenin-7,4^′^-dimethylether	5.00-1000.00	Y = 165X + 375	0.9861	1.50	5.00

**Table 4:** Experimental concentration values (ng/mL) obtained for intra-day precision (n = 6) and inter-day precision (n = 18) in UFLC-MS/MS analysis for the nigh compounds in the extract solution of samples

**Compounds**	**Precision RSD (%)**		**Repeatability (RSD, %, n = 6)**	**Stability (RSD, %, n = 6)**	**Recovery (%, n = 3)**	
	**Intraday (n = 6)**	**Interday (n = 6)**			**Mean**	**RSD**
Nootkatone	2.37	1.71	3.81	3.74	104.82	3.52
Yakuchinone A	3.32	4.81	3.56	5.11	104.13	4.69
Yakuchinone B	2.77	3.39	2.90	3.79	95.97	3.79
Oxyphyllacinol	4.01	2.49	3.60	2.43	103.14	4.00
Tectochrysin	1.61	2.86	2.51	4.78	105.09	3.70
Izalpinin	1.61	3.13	2.36	3.36	103.56	5.63
Chrysin	1.93	3.64	3.02	4.41	98.87	3.66
Kaempferide	2.29	2.67	3.40	2.20	101.62	3.70
Apigenin-7,4^′^-dimethylether	4.36	3.91	4.98	3.92	95.07	1.69

### Quantitative analysis of samples

**Figure 3:**
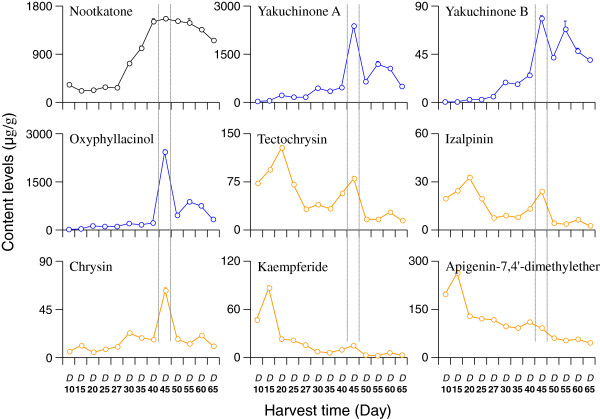
**The content levels of nine compounds of different harvest time fruits of *Alipina oxyphylla *were assessed under the optimized extraction and UFLC-MS/MS analytical conditions, and the 45-day culture fruit had the highest levels.**

The proposed UFLC-MS/MS method was subsequently applied to determine nine chemical markers including nootkatone (**1**), yakuchinone A (**2**), yakuchinone B (**3**), oxyphyllacinol (**4**), tectochrysin (**5**), izalpinin (**6**), chrysin (**7**), kaempferide (**8**) and apigenin-7,4′-dimethylether (**9**) from the 10-day to 65-day samples of fruits. The results (Table 5 and Figure 3) showed that three kinds of components accumulated with the growth time. But there were remarkable differences in their contents at different harvest time. For example, there was few nootkatone before 27 days in fruits. With the growth of fruit, the content of nootkatone entered a rapid growth phase and reached the maximum (mean value as 1.557 mg/g) at 45-day. From 45-day to 55-day, the content of nootkatone in fruits showed a little downward trend from 1.557 mg/g to 1.160 mg/g. For the three diphenylheptanes compounds, the content of every compound was very low before 40-day but at 45-day all their contents increased sharply to the maximum (mean value as 2.401 mg/g for yakuchinone A, 0.0795 mg/g for yakuchinone B and 2.465 mg/g for oxyphyllacinol). After 45-day, the contents of three diphenylheptanes compounds declined to 503 μg/g for yakuchinone A, 40 μg/g for yakuchinone B and 353 μg/g for oxyphyllacinol at 65-day. The flavones compounds exhibited another characteristics. Most of flavones achieved the maximum content before 30 harvest time. The tectochrysin had the maximum content of 128 μg/g at 20-day, the izalpinin of 33.2 μg/g at 20-day, the kaempferide of 264 μg/g at 15-day and apigenin-7,4′-dimethylether of 86.7 μg/g at 15-day. Only the content of chrysin achieved the maximum of 62.9 μg/g at 45-day. The differences of content variation between flavones with other two kinds of compounds might indicate the biosynthetic pathway of flavones was differentiated with the other two types of phytochemicals.

**Table 5:** Chemical contents in fruits of different harvest time

**Harvest time(day)**	**Nootkatone**	**Yakuchinone A**	**Yakuchinone B**	**Oxyphyllacinol**	**Tectochrysin**	**Izalpinin**	**Chrysin**	**Apigenin-7,4'-dimethylether**	**Kaempferide**
10	331 ± 25	38.9 ± 3.9	0.58 ± 0.02	28.9 ± 2.8	73.2 ± 3.5	20 ± 0.7	5.49 ± 0.19	197 ± 8	47.0 ± 2.3
15	211 ± 4	56.3 ± 2.4	0.76 ± 0.05	48.2 ± 3.3	94.6 ± 1.4	24.8 ± 0.4	11.2 ± 0.5	264 ± 9	86.7 ± 2.7
20	232 ± 7	223 ± 18	2.98 ± 0.16	150 ± 3	128 ± 4	33.2 ± 1.3	4.89 ± 0.13	129 ± 5	23.2 ± 1.2
25	279 ± 11	170 ± 8	2.62 ± 0.13	121 ± 4	71.6 ± 3.4	20.1 ± 0.9	7.6 ± 0.32	122 ± 4	21.3 ± 0.8
27	267 ± 9	174 ± 16	5.78 ± 0.31	126 ± 4	33.5 ± 1.2	7.91 ± 0.29	9.83 ± 0.52	118 ± 4	15.3 ± 0.7
30	726 ± 8	441 ± 16	19.2 ± 0.7	231 ± 11	40.8 ± 1.7	9.47 ± 0.19	22.6 ± 0.7	97.4 ± 4.6	7.18 ± 0.41
35	1003 ± 48	358 ± 12	17.1 ± 0.8	184 ± 7	34.5 ± 1.6	8.39 ± 0.39	18.6 ± 0.9	92.3 ± 3	5.83 ± 0.27
40	1506 ± 51	470 ± 15	25.7 ± 2.1	243 ± 9	57.8 ± 2	13.6 ± 0.4	16.6 ± 0.7	111 ± 2	9.75 ± 0.38
45	1557 ± 38	2401 ± 37	79.5 ± 2.7	2465 ± 79	80.8 ± 3.6	24.2 ± 1.2	62.9 ± 2.6	91.2 ± 1.1	15.1 ± 0.6
50	1515 ± 17	648 ± 36	42.4 ± 1.4	487 ± 20	17.7 ± 0.8	4.34 ± 0.19	17.6 ± 0.7	61 ± 4	3.24 ± 0.13
55	1486 ± 71	1202 ± 93	69.3 ± 8	912 ± 41	17.1 ± 1.9	4.31 ± 0.33	12.7 ± 1.1	53 ± 4.2	2.3 ± 0.09
60	1357 ± 24	1065 ± 39	48.6 ± 2.8	777 ± 15	28.8 ± 1.4	6.56 ± 0.46	20.5 ± 1.1	56.3 ± 1.5	6.18 ± 0.11
65	1160 ± 10	503 ± 10	40 ± 1.4	353 ± 17	15.5 ± 0.5	2.86 ± 0.06	10.5 ± 0.5	45.5 ± 3.9	2.78 ± 0.13

## Conclusion

In this paper, an UFLC-MS/MS method was proposed and validated as a reliable and powerful technique for simultaneous qualification and quantification of nine compounds in the fruits of *A. oxyphylla* within 15 min. This method was applied to investigate the contents of nine compounds harvested at different time. The data showed there were remarkable differences at the harvest time and contents of the chemical markers among three types of chemical. The sesquiterpene compound, i.e., nootkatone accumulated with the growth of plant. The contents of three diphenylheptanes compounds showed a sharp increase at the ripe fruit of 45-day. The flavonoids presented another rule that had the maximum contents before 30-day. These results suggested that the sesquiterpene and diphenylheptane compounds might two important components for the fruit.

The results mentioned above showed that our work could offer a general analytical method for the quality control of the fruits of *A. oxyphylla* and also could provide some useful information for rational harvest periods of *A. oxyphylla* resources.

## Abbreviations

UFLC: Ultra fast liquid chromatography; UFLC-MS/MS: Ultra fast liquid chromatography coupled with mass spectrometry and mass spectrometry detector; HPLC: High performance liquid chromatography; HPLC–PDA-MS: High performance liquid chromatography coupled with photo diode array and mass spectrometry detector; RSD: Relative standard deviations.

## Competing interests

The authors declare that they have no competing interests.

## Authors’ contribution

LYH and CF carried out the orthogonal design optimization studies, participated in the drafted the manuscript. WJF carried out the extraction of *A. oxyphylla*. WY participated in the design of the study and performed the statistical analysis. ZJQ and GT conceived of the study, and participated in its design and coordination and helped to draft the manuscript. All authors read and approved the final manuscript.
